# A P2P Botnet detection scheme based on decision tree and adaptive multilayer neural networks

**DOI:** 10.1007/s00521-016-2564-5

**Published:** 2016-10-03

**Authors:** Mohammad Alauthaman, Nauman Aslam, Li Zhang, Rafe Alasem, M. A. Hossain

**Affiliations:** 1 Department of Computer Science and Digital Technologies, Faculty of Engineering and Environment, Northumbria University, Newcastle upon Tyne, NE1-8ST UK; 2 Department of Electrical Engineering, Faculty of Engineering, Imam Mohammad Ibn Saud Islamic University, Riyadh, Saudi Arabia; 3 Information Technology Institute, Anglia Ruskin University, Bishop Lane, Chelmsford, CM1 1SQ UK

**Keywords:** P2P Bot, Multilayer neural network, CART algorithm, TCP protocol, C&C, Resilient back-propagation

## Abstract

In recent years, Botnets have been adopted as a popular method to carry and spread many malicious codes on the Internet. These malicious codes pave the way to execute many fraudulent activities including spam mail, distributed denial-of-service attacks and click fraud. While many Botnets are set up using centralized communication architecture, the peer-to-peer (P2P) Botnets can adopt a decentralized architecture using an overlay network for exchanging command and control data making their detection even more difficult. This work presents a method of P2P Bot detection based on an adaptive multilayer feed-forward neural network in cooperation with decision trees. A classification and regression tree is applied as a feature selection technique to select relevant features. With these features, a multilayer feed-forward neural network training model is created using a resilient back-propagation learning algorithm. A comparison of feature set selection based on the decision tree, principal component analysis and the ReliefF algorithm indicated that the neural network model with features selection based on decision tree has a better identification accuracy along with lower rates of false positives. The usefulness of the proposed approach is demonstrated by conducting experiments on real network traffic datasets. In these experiments, an average detection rate of 99.08 % with false positive rate of 0.75 % was observed.

## Introduction

Internet services are increasing in popularity, and many new online services appear every day. The use of online services leads to a massive volume of online financial transactions, where sensitive information is exchanged via the Internet. The attacker’s interest is converted from curiosity to financial benefit. Attackers use different malware to achieve their goals. Among the various forms of malware, Botnet is considered to be the most serious means for conducting online crime [1]. However, financial profit is the goal of Botnets creation and development by attacker [2].

**Fig. 1:**
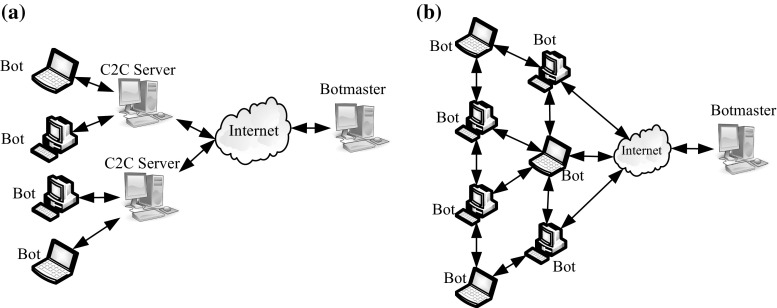
Structures of the Botnet. **a** Centralized structure, **b** decentralized structure

A Botnet is a network of compromised computers (Bots) remotely managed by an attacker (Botmaster). A Botnet can be ordered to perform various malicious activities, such as sending spam emails, phishing, click fraud, DDoS and spreading malicious software. To effectively administer a Botnet, the Botmaster constructs an infrastructure of a communication channel to send commands to the Bots and to receive results from them [3]. This communication channel is known as the command and control (C&C) channel. The main difference between a Botnet and other malware is the infrastructure used in the C&C [4]. In contrast to other malware that is used to perform malicious behaviour individually, a Botnet works as a group of infected hosts based on the C&C communication channel. A Botnets network can be classified into two main categories based on the C&C infrastructure: centralized and decentralized C&C [5]. In centralized Botnets, the Botmaster normally uses the C&C server to send a command to the Bots as shown in (Fig. 1a).

Due to its simplicity, the centralized Botnet is widely used by many Botnet families. The most famous approaches are the Internet Relay Chat (IRC) and Hypertext Transfer Protocol (HTTP) Botnet. However, the main limitation of the centralized Botnet is its single point of failure C&C server. A shutdown of the C&C server would result in the loss of communication between the Bots and the Botmaster [6]. In order to avoid the weakness of a single point of failure, Botnet attackers have recently started to build Botnets based on decentralized C&C infrastructures such as the P2P Botnet [7] and P2P model was adopted by many types of Botnet, e.g. Storm Bot, Conficker Bot and Waledac Bot [8].

A P2P Botnets are a new class that has replaced the old centralized IRC/HTTP-based Botnet to avoid a single point of failure and avoid detection during C&C connection. Due to the distributed network structure of P2P systems, all peers in the network work as a Bot (client) and C&C (server) at the same time. In this case, the Botmaster plays the main role by sending commands to any infected peers to execute any order or requesting information at any time (see Fig. 1b).

The life cycle of the P2P Botnet consists of four primary phases, namely: initial infection, peer propagation, secondary injection and attack [7]. Firstly, Bot code is created to insert on an end-user computer by different techniques such as web download, vulnerability exploitation, mail attachments, automatically scan, exploit and compromise, traditional file-based viruses [9]. Secondly, the Bot tries to connect with other Bots on infected hosts based on its own hard-coded peer list. Thirdly, the Bot downloads the latest update of the Bot code through the C&C channel, which will update it for future tasks. In this phase, a host is considered to be a Bot in Botnet network. Finally, the Bot initiates malicious activities such as spam or phishing emails, distributed denial-of-service attacks (DDoS), stealing information and scanning activities.

Despite many research efforts, the P2P Botnet detection remains a significant challenge for the researchers. Firstly, the traffic of P2P Botnet is similar to normal traffic; and secondly, many P2P Botnets, such as Storm and Waledac, use encryption algorithms that make methods based on packet inspection ineffective. Furthermore, there is no central server in P2P Botnets and in addition Bots contact other peers using random ports [10].

The main aim of this research is to develop P2P Bot detection approach based on traffic reduction technique. The approach proposed in this research has the following characteristics. It detects Bots during the propagation phase before any malicious action has been taken. Furthermore, it does not require deep packet inspection (DPI) analysis for signature matching and does not need to analyse the entire network traffic. It detects Bots independent of port numbers, IP addresses and host characteristics. In summary, we made the following contributions:

- A network traffic reduction approach that has been designed will be able to increase the performance of the proposed framework.
- A connection-based detection mechanism is independent of payload and uses only the information obtained from the header of TCP control packet. Thus, it does not need deep packet inspection and cannot be confused with payload encryption techniques.
- Adopting the classification and regression trees to select the important connection features in order to decrease the size and dimensionality of the dataset.
- Detection of P2P Bot traffic on the network and discriminating it from legitimate network traffic.

The rest of this paper is organized as follows. Section 2 briefly reviews work relevant to P2P Botnet detection approaches. The proposed approach is then described in Sect. 3. Section 4 presents the experimental results, and finally the conclusions and suggestions for future work are presented in Sect. 5.

## Relevant work

In recent years, there has been an increasing interest in techniques for Bot detection and prevention. While it may be important to learn how a Bot infects the computers, it is more critical to detect the infected machine before it is exploited to launch malicious activities. Several approaches to detect Botnets have been developed. These approaches can be classified into signature-based, anomaly-based, DNS-based and data mining techniques [11]. Another researchers such as Han et al. [5] classified P2P Botnet detection systems into three general types: data mining, machine learning and network behaviour and traffic analysis. What is more, Zeidanloo et al. [12] in their research classify the Botnet detection system as honeynets or intrusion detection systems (IDS) and also divided the IDS system into three subgroups as following: anomaly-based, specification-based and signature-based. In addition, the Botnet detection system can be classified based on the installation point as a host-based, network-based and hydride systems. Lu et al. [3] have classified the Botnet detection techniques on the basis of machine-learning type supervised and unsupervised Botnets detection.

A recent study in the field of P2P Botnet detection by Babak et al. [13] proposed PeerRush, which uses a one-class classification approach to classify various types of normal and abnormal P2P traffic. One-class classifier including KNN, Parzen and Gaussian data description classifiers [14] is used. An application profile is initially created by learning traffic samples of known P2P applications. Moreover, features such as interval delays between packets and flow duration are used to classify P2P applications. This approach achieves high accuracy rate in classifying P2P applications depending on the features selected. On the other hand, this method does not show clearly how to detect P2P Botnet, and also detection can be easily avoided by changing the delay between packets.

In [15], Garg et al. presented a several machine-learning algorithms such as nearest neighbour, Naive Bayes and J48. These have been analysed for the detection of P2P Botnets using various network traffic features. The results show that the accuracy of the classifiers trained using the nearest neighbour and J48 is good. However, the detection of legitimate traffic is very weak.

Jiang and Shao [16] present a method that focuses on the C&C traffic of P2P Bots regardless of how they perform their malicious activity. This method develops a detection mechanism based on a Bots which exhibit connection flow dependency with other Bots. According to the flow dependency behaviour, this approach uses single-linkage hierarchical clustering mechanism to differentiate between P2P Bots and normal hosts. This method was built based on the similarity of Botnet traffic, so this approach will fail to detect the Botnet, which uses the irregularity of traffic flow such as Storm Bot [17]. Furthermore, it has a limitation to identify individual Bot behaviour.

EFFORT [18] is a host-based detection approach which collects information related to Bot’s characteristics at client and network level. It then correlates Bot-related information by monitoring local computer activity such as keystrokes and connections with other computers. The main advantage of this method is that it does not depend on protocol and communications topology. In addition, it is able to detect Bots that are using encryption techniques to hide the malicious behaviour. The major limitations of this method are critical to evasion techniques such as fast-flux, and it cannot prove as real-time detection approach.

Masud et al. [19] introduced an approach to Botnet detection based on the observation that a Bot has many reaction patterns which are different from those of humans. This method can detect Bots by correlating incoming packets with outgoing packets, new outgoing connections and application start-ups in hosts. Several machine-learning algorithms such C4.5 decision tree, support vector machine, Naive Bayes, Bayes network classifier and boosted decision tree [20] were compared and evaluated in the detection of IRC Botnet. The result of the classifiers evaluation shows that all machine-learning algorithms achieve 95 % detection rate, less than 3 % false positive rate and under 5 % false negative rate. The greatest overall performances were reached by a boosted decision tree. However, one major drawback of this approach is that it cannot detect Botnets that use encrypted communication because it needs to access the content of the payload packets. On the other hand, the method has been tested on IRC Bots, therefore its ability to detect modern types of malware such as P2P Bots is not known.

Zhang et al. [21] introduced a P2P Botnet detection system that can identify stealthy P2P Botnets. The proposed approach focuses on identifying Bots based on the monitoring of C&C traffic. They extract four features for each traffic flow including the number of bytes received and sent and number of packets received and sent. Hierarchical clustering [22] and BIRCH algorithm [23] are used to cluster network flow. Furthermore, the approach is independent of payload signatures and has also achieved a high rate of detection both malicious and legitimate hosts, with the FPR of 0.2 % and TPR 100 %. Although this system can detect Botnets regardless of how they perform malicious activities, it focuses only on P2P Botnet and cannot detect other types such as IRC or HTTP Bots. However, the proposed technique is vulnerable with some of the evasion methods such as flow disturbance packets and by using the DGA and fast-flux algorithms as a communications facility in order to provide C&C a high level of privacy.

Liao et al. [24] used a methodology based on packet size to distinguish between P2P Botnet traffic and legitimate P2P traffic. They presented the following observations. Firstly, P2P Bots tries to update information for other Bots rather than staying idle. Secondly, the Bot mainly transmits data with a minimum rate of connections. Bayesian networks, Naïve Bayes and J48 are used to classify network traffic. Furthermore, the accuracy rates for the three algorithms are 87, 89 and 98 %, respectively. However, it was found that the size of P2P Botnet packets is smaller than that of any other P2P applications.

The detection system introduced by Fedynyshyn et al. [25] uses a host-based approach to detect Bots using the property of temporal persistence. They utilized a J48 classifier and a random forest algorithm for sorting various kinds of Botnet infection categorized according to their C&C model (HTTP, IRC and P2P). Moreover, they found similarities in C&C structures for different categories of Bots that are different from those of legitimate network traffic.

In 2014, Zhang et al. [26] introduced an approach based on their previous research in 2011 [21] to enhance the performance of the system scalability and increase the efficiency. The method includes two main phases which are: (1) recognizes all machines that are possibly involved in P2P connections and extracts statistical fingerprints from profile P2P traffic, (2) analyses the traffic of P2P hosts to classify them as P2P bots or legitimate P2P hosts. In the experiment, P2P applications such as eMule, LimeWire, Skype and BitTorrent were run on various machines to generate legitimate traffic. Besides, Waledac and Storm were run in a controlled environment to generate malicious network traffic. By using hierarchical clustering of P2P flows the approach capable of distinguishing legitimate P2P traffic from P2P Botnet traffic with 100 % detection rate and 0.2 % false positive detection rate. The significant advantage of the method is that it is efficient to distinguish Bots traffic which is overlap within legitimate host traffic with high detection rates.

Zhao and Traore [27] introduced a P2P Botnet detection technique based on recognizing the malicious behaviour of fast-flux networks. They calculate metrics of features from captured network traffic which are used to identify Botnet traffic. However, the approach through using decision tree algorithm achieves high accuracy rates.

In the proposed approach, a decision tree is utilized as a feature set reduction mechanism to exclude insignificant features for the endeavour of downsizing the quantities of data necessary to acquire better classification accuracy rate, learning rate and reducing in computational time. It includes a unified method which incorporates classification and regression trees (CART) [28] and a multilayer feed-forward neural network with resilient back-propagation algorithm [29] for the use of P2P Bot detection.

The proposed system uses the header of TCP control packets to bypass the encrypted network traffic and reduce the number of packets that will enter to the detection system. Moreover, focusing on the connection behaviour will help the detection system to recognize the Bot behaviour at an earlier stage when the Bot propagates and tries to contact with other peers to find new updates. Furthermore, the proposed feature sets are estimated for every connection in the network to detect any single infect machine. To the best of our knowledge, this is the first time that connection-based features are used in P2P Bot detection. As the features are extracted from packets headers, they do not rely on the packet payloads. With this characteristic, our detection approach will not be affected by traffic encryption. Furthermore, the feature sets help the detection system to identify P2P Bot infects even single host in the network.

## Proposed approach

**Fig. 2:**
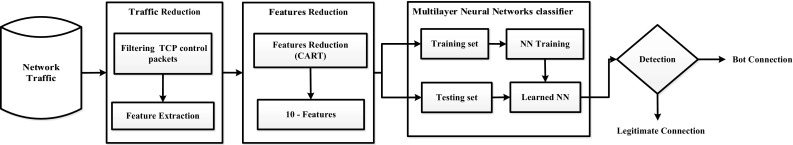
Block diagram of the proposed system

The proposed framework relies on two fundamental concepts. Firstly, it is passively monitoring network traffic [12]. Secondly, it utilizes the fact that Bots during the propagation phase will show frequent communication behaviours with their C&C servers/peers in order to discover other peers and receive the latest update of tasks due to their pre-programmed nature [30, 31]. Bots are different from other malware in that they work as a group and they primarily need a communication channel to coordinate their malicious activities. These connections are described as the way by which the Botmaster communicates with his Bots [9]. The proposed P2P Bot detection uses a multilayer feed-forward neural network with adaptive learning rate, since most well-known P2P Bots communicate using TCP connections [32] such as Waledac Bot [33], Storm Bot [34], Conficker Bot [35] and Zeus Bot [36, 37]. Therefore, in this paper features related to TCP connection have been extracted based on TCP control packets. To increase the learning rate, a resilient back-propagation algorithm is used. The resilient back-propagation is considered to be the best algorithm, measured in terms of convergence speed, accuracy, robustness and with respect to training parameters [29]. Figure 2 shows a block diagram of the proposed system.

### Network traffic reduction

Network traffic reduction for detection of malicious activities is essential for managing enormous amounts of network traffic where resources are restricted (e.g. memory, hard disk). The most difficult part of this process is to identify the behaviour of network traffic by inspecting only a small number of packets per flow. Therefore, this research introduces a new traffic reduction technique to facilitate the deployment of Bot detection systems on high-speed networks.

The most of the existing Botnet detection systems [38–41] rely on deep packet inspection (DPI) to analyse packet content, which is computationally expensive and inefficient to recognize unknown payload signature [42]. In DPI, the system is assumed to have access to the payload of every packet. This technique can be notably accurate when the payload is not encrypted. However, the majority of new malware generation applies evasion methods such as encryption of payload, protocol encapsulation and obfuscation [43].

Furthermore, examining all packets on a high-speed network is an expensive task because the speed of networks and the amount of the packet transferred via networks are increasing daily. Thus, the detection system which applies DPI may suffer from efficiency bounded on processing a large volume of traffic from high-volume or high-speed networks [42]. The goal of our work is to increase the effectiveness of the detection systems by decreasing the volume of traffic to be analysed, without affecting the accuracy of the detection process. To achieve this goal, a novel traffic reduction is proposed for a Bot detection framework by selecting only TCP control packets. The framework can efficiently and effectively reduce the amount of traffic that will be entering into the detection system. To the best of our knowledge, this first P2P Bot detection approach applies reduction technique to achieve the efficiency on Botnet detection domain.

In this study, a filtration of TCP control traffic packets is used to reduce the volume of network traffic as well as to increase the performance of the proposed approach. The filtering includes two steps: filtering all traffics related to the TCP protocol; then extracting the TCP control packet SYN, ACK, FIN and RST. Algorithm 1 shows the process of reduction network traffic from network traces (.PCAP files). In Line 2, an array of TCP_Control_Packets_list is initialized. By iterating over the packets, new packets are added to the array of (TCP_Control_Packets_List) from Line 3 to 15 till the last packet in the file is reached. Line 4 examines for TCP packet header, and Line 5 selects packets with no payload data. Line 6 gets the packet header. From Line 7 to 10, the code reads the packet, which is TCP, and extracts the packets having SYN, ACK, FIN and RST flags.

In summary, the network traffic reduction algorithm 3.1 includes six rules to pick the desired packets:

- *Rule 1* (*R1*) Packet contents SYN flag.
- *Rule 2* (*R2*) Packet contents SYN–ACK flag.
- *Rule 3* (*R3*) Packet contents ACK flag.
- *Rule 4* (*R4*) Packet contents FIN–ACK flag.
- *Rule 5* (*R5*) Packet contents Rest–ACK flag.
- *Rule 6* (*R6*) Packet contents Rest flag.

### Feature extraction

In the features extraction phase, the features that are important in detecting the Bot’s malicious behaviour are extracted, and these features are collected in 29-tuple attributes based on 30-s connections. These features are extracted based on the definition of a connection as a group of packets exchanged between two different hosts, which are identified by the 4-tuple (source IP address, destination IP address, source port and destination port). In our proposed method, all features are extracted directly from the control packet header, rather than previous approaches using deep inspection of packet payload content, e.g. [3, 44–46]. Therefore, performance is increased, and the use of the system resources such as memory and computations in the processor is reduced. Table 1 shows the 29 features created in the proposed connections-based P2P Bot detection approach. These features are generated from a 30-s connection and are composed of a feature vector to represent the features of a 30-s connection.

**Table 1:** Selected features of network traffic connections

Features	Description
F1	Number of control packets per flow in a given time interval
F2	Number of control packets transmitted per flow in a given time interval
F3	Number of control packets received per flow in a given time interval
F4	Number of transmitting bytes per flow in a given time interval
F5	Number of received bytes per flow in a given time interval
F6	Number of transmitted SYN packets per flow in a given time interval
F7	Number of received SYN packets per flow in a given time interval
F8	Number of transmitted ACK packets per flow in a given time interval
F9	Number of received ACK packets per flow in a given time interval
F10	Number of transmitted duplicate ACK packets per flow in a given time interval
F11	Number of received duplicate ACK packets per flow in a given time interval
F12	Average length of transmitted control packets per flow in a given time interval
F13	Average length of received control packets per flow in a given time interval
F14	Average length of control packets per flow in a given time interval
F15	Number of transmitted failed connection per flow in a given time interval
F16	Number of received failed connection per flow in a given time interval
F17	Number of transmitted ACK packets have a sequence one per flow in a given time interval
F18	Number of received ACK packets have a sequence one per flow in a given time interval
F19	Number of transmitted SYN–ACK packets per flow in a given time interval
F20	Number of received SYN–ACK packets per flow in a given time interval
F21	Total number of bytes per flow in a given time interval
F22	Ratio of incoming control packets per flow in a given time interval
F23	Ratio of average length of outgoing packets over the average length of control packets per flow in a given time interval
F24	F6–F20
F25	Number of transmitted FIN–ACK packets per flow in a given time interval
F26	Number of received FIN–ACK packets per flow in a given time interval
F27	Number of transmitted RST–ACK packets per flow in a given time interval
F28	Number of received RST–ACK packets per flow in a given time interval
F29	Average time between an attempt to create connection per flow in a given time interval

### Features reduction

Feature reduction is the technique of reducing the number of attributes, with the purpose of eliminating those features from the learning algorithm that have a small influence on the classification problem [47]. Feature reduction is used to decrease the ‘over-fitting’ problem [48] and is important to overcome the imbalance dataset problem [49]. Therefore, the quality of the feature reduction mechanism is one of the most important factors that affect the accuracy of the classification algorithm.

In this study, the aim of feature reduction is to choose a suitable subset of features, which will improve neural network performance and decrease the complexity of a classification model without significantly decreasing accuracy rates. In this study, a classification and regression tree (CART) is employed as the feature reduction approach used to eliminate worthless features, with the aim of reducing the quantity of data needed to obtain better rates of neural network learning and classification accuracy.

The decision tree produced by the CART algorithm consists of two types of node: internal nodes with two children and leaf nodes without children. Any internal node is associated with a decision function to indicate which node to visit next. To begin the construction of the tree, the training samples that contain a set of features and their class labels are required. The training set is recursively divided into smaller subsets during the construction of the tree. Based on the decision matrix from the distribution of classes in the training set, each resulting node is assigned a predicted class. The test at internal nodes is determined based on a measure of impurity to select which feature and which threshold values are selected. The best-known measure of impurity for CART is entropy impurity which is given by.

1 $$ E\left( t \right) = - \mathop \sum \limits_{j}^{C} p\left( {\frac{j}{t}} \right)\log_{2} p\left( {\frac{j}{t}} \right) $$

where *E* (*t*) is the entropy impurity at node *t*, $p\left( {\frac{j}{t}} \right)$ is the relative frequency of class j at node *t*, and *c* is the number of classes.

The best value of the split node (*t*) is chosen from a set of all values splitting (*X*), so that the maximum drop in impurity is a difference between impurity at the root node and impurity at the children nodes:

2 $$ \Delta E\left( {X,t} \right) \, = \, E\left( t \right) \, {-} \, \left( {P_{\text{L}} E\left( {t_{\text{L}} } \right) \, + \, P_{\text{R}} E\left( {t_{\text{R}} } \right)} \right) $$

where ∆*E*(*X*, *t*) is the drop of impurity, *E*(*t*_L_) and *E*(*t*_R_) are the impurities of the left and right branch nodes, *P*_L_ and *P*_R_ are the percentages of objects go to the left (*t*_L_) or right (*t*_R_) child nodes. Table 2 provides a ranking of features’ importance selected by the CART algorithm. The features F3, F13, F23, F21, F14, F29, F12, F1, F4 and F15 have the best discrimination of the connections behaviour, whereas the features F2, F7, F9, F11, F16, F18, F19, F20, F22, F24, F25, F26, F28 have no discrimination between legitimate and malicious connections.

**Table 2:** Features importance ranking by CART and ReliefF

CART algorithm		ReliefF algorithm	
Feature	Importance	Feature	Importance
F3	100	F27	0.08668
F13	69.77551	F25	0.031391
F23	58.82751	F15	0.026481
F21	14.94384	F6	0.026306
F14	2.900449	F22	0.02497
F29	0.794777	F24	0.024034
F12	0.384592	F29	0.023641
F1	0.120902	F23	0.016308
F4	0.082941	F26	0.013599
F15	0.069167	F19	0.011077
F6	0.012049	F14	0.008974
F5	0.01191	F13	0.004725
F27	0.01153	F12	0.004475
F10	0.000515	F28	0.004378
F8	3.81E−06	F18	0.004236
F17	6.12E−09	F3	0.003006
F2	0	F1	0.002928
F7	0	F9	0.002817
F9	0	F20	0.002746
F11	0	F4	0.002391
F16	0	F8	0.002162
F18	0	F2	0.002123
F19	0	F21	0.001838
F20	0	F17	0.001292
F22	0	F11	0.00126
F24	0	F10	0.001083
F25	0	F5	0.00054
F28	0	F16	0
F26	0	F7	0

ReliefF is generally utilized in the data pre-processing phase as a feature selection approach. The key idea of the ReliefF is to evaluate the quality of attributes according to how well their values discriminate between the instances that are near to each other [50]. The ReliefF algorithm essentially consists of three important parts: firstly, estimation of the nearest miss and nearest hit; secondly, estimation of the weight of a feature; thirdly, return a ranked list of features. The pseudo code of the ReliefF algorithm is given in Algorithm 2 [51]. Table 2 shows the important ranking of features estimated by the ReliefF algorithm.

The principal component analysis (PCA) is a feature selection and, to be precise, is also a feature reduction approach. PCA reduces the initial number of features to a smaller number of uncorrelated features, which are calculated as the linear combination of the original ones [52]. For instance, each principal component in PCA is the linear combination of the variables that gives a maximized variance [53]. The mathematics behind PCA is briefly described here.

Given an *M* × *N* matrix, *X*, where *M* is the number of attributes and *N* is the number of samples. The mean *m* of the training samples (corresponding to a column vector *N*) is given:

3 $$ m = \frac{1}{M}\mathop \sum \limits_{i = 1}^{M} xi $$

Centralize the matrix *X* by subtracting *m* from each *xi*.

4 $$ yi = xi - m $$

Further, the covariance matrix is estimated by

5 $$ {\text{cov}} = \frac{1}{M}\mathop \sum \limits_{i = 1}^{M} yi \cdot yi^{T} $$

Calculate the eigenvectors and eigenvalues of the covariance matrix, and then select top *k* eigenvectors that correspond to the top k eigenvalues. The top *k* principal components are picked that retains 95 % (in the WEKA machine learning [54]) of the data’s overall variance.

PCA and ReliefF algorithms have also been used for the reduction of the feature set from the same set of features, and a comparison is made of the performances of these algorithms. The higher ten important features selected by each algorithm are summarized in Table 3.

**Table 3:** Feature reduction with the CART, PCA and ReliefF algorithms

Feature selection algorithm	Features number	Feature list
CART	10	F3, F13, F23, F21, F14, F29, F12, F1, F4, F15
PCA	10	Linear combination of features
ReliefF	10	F27, F25, F15, F6, F22, F24, F29, F23, F26, F19

### Neural network

The neural network is currently a subject of wide interest. It has robust capabilities for nonlinear system identification and control due to an inherent ability to approximate an arbitrary nonlinear problem [55–57]. The basic architecture of neural network includes input layer, one or many hidden layers and output layers. Moreover, every layer contains a specific number of neurons. The result from any neuron is used as input to another neuron in the next layer. The link between neurons has an associated weight. A neural network is trained by giving input and target sets repeatedly. Each input is given, and the network computes an output. The neural network outputs are used to determine the accuracy of results and whether the network is wrong or right. Whenever wrong, the network has improved the weight using a back-propagation based on the difference between the output and desired target of the neural network. After each iteration, the network reduces the error between output and target.

For the purposes of the present study, the neural network is trained with a resilient back-propagation learning algorithm, where the use of this algorithm is to minimize the damaging effects of the volumes of fractional derivatives. The sign of the derivative is only used to locate the trend of the weight update, whereas the volume of the derivative has no negative role overweight update. The size of the weight change is solely determined by the following formula [29]:

6 $$ \Delta w_{ij}^{\left( t \right)} = \left\{ {\begin{array}{l} { - \Delta_{ij} \left( t \right), \quad {\text{if}} \quad \frac{{{\partial }E\left( t \right)}}{{{\partial }w_{ij} }} \; > \;0  } \\ { + \Delta_{ij} \left( t \right), \quad {\text{if}} \quad \frac{{{\partial }E\left( t \right)}}{{{\partial }w_{ij} }} \; < \;0} \\ {0, \quad \quad  {\text{else}}             } \\ \end{array} } \right. $$

where $\Delta w_{ij}^{\left( t \right)}$ is the change in weight between input layer and hidden layer by the current iteration (*t*) and $\frac{\partial E\left( t \right)}{{\partial w_{ij} }}$ denotes the partial derivative with respect to each weight. Once the weights are calculated, the new weight updated value is determined. This is accomplished with the following formula:

7 $$ \Delta_{ij}^{\left( t \right)} = \left\{ {\begin{array}{l} {\eta^{ + } \cdot \Delta_{ij} \left( t \right), \quad {\text{if}}\quad  \frac{{{\partial }E\left( {t - 1} \right)}}{{\partial w_{ij} }} \cdot \frac{{{\partial }E\left( t \right)}}{{{\partial }w_{ij} }} \; > \;0  } \\ {\eta^{ - } \cdot \Delta_{ij} \left( t \right), \quad {\text{if}}\quad \frac{{{\partial }E\left( {t - 1} \right)}}{{{\partial }w_{ij} }} \cdot \frac{{{\partial }E\left( t \right)}}{{{\partial }w_{ij} }}  \; < \;0} \\ {\Delta_{ij} \left( {t - 1} \right),\quad {\text{else}}                               } \\ \end{array} } \right. $$

where $\Delta_{ij}^{\left( t \right)}$ denotes the updated value for the current iteration *t*, *η* ^+^ is the positive step value which is typically 1.2, and *η* ^−^is the negative step value which is typically 0.5.

The neural network classifier proposed in this study contains ten input and two output parameters. To avoid overfitting by using too many hidden layers, the method proposed in a previous study [28] is used to determine the number of neurons in hidden layers.

## Experimental results and analysis

### Dataset

Two datasets that contain malicious and non-malicious traffic were obtained for use in evaluating our proposed system. The first is the ISOT dataset [58] that contains malicious traffic from the French chapter of the Honeynet Project involving the Waledac and Strom Bots. It also contains non-malicious traffic collected from the Traffic Lab at Ericsson Research in Hungary and from the Lawrence Berkeley National Laboratory (LBNL). The second is the ISCX dataset [59] which includes normal activity and non-malicious traffic. Table 4 shows the samples of network traces used in the experiment and the evaluation of the proposed model.

**Table 4:** Datasets selected

Traffic type	Duration (s)	Number of packets	Number of control packets	Number of connections
Storm Bot traffic	3115	128,241	64,551	5423
Waledac Bot traffic	605	118,379	49,536	4535
Normal traffic (ISCX)	9511	419,659	165,218	11,088
Normal traffic (LBNL)	126,273	564,999	166,308	12,001

### Experiment

To generate an experimental dataset with both P2P Botnets traffic and normal legitimate traffic, the trace (.PCAP) files were replayed using the TcpReplay tool on the same network interface card; then the network traffic was captured via Wireshark for evaluation. After that, a MATLAB script was used to generate connections and to extract features from PCAP file. Connections were then labelled in two classes of Bot and normal connections. In this work, a network connection is defined as 4-tuple, with source IP address, source port number, destination IP address and destination port number, which have transferred to at least one packet in both directions.

### Performance evaluation and results

In order to evaluate the rate of accurate detection, *N*-fold cross-validation is used to estimate the error rate of classifiers. In *N*-fold cross-validation, the dataset is partitioned randomly into N samples and evaluations run for N iterations. In each iteration, *N*−1 samples are selected for training and the final sample is used to evaluate the accuracy of the classifier. *N* = 10 was selected in conducting the experiments. The performance of the proposed model is compared with that of the PCA and ReliefF algorithm as others feature selection approaches. To evaluate the performance of the neural network recognition system, measures such as false positive rate, true positive rate, accuracy, precision, recall and the *F*-measure are calculated as follows:

- *True positive* (*TP*) the number of malicious behaviours correctly detected as malicious activities.
- *True negative* (*TN*) the number of normal behaviours correctly detected as normal activities.
- *False positive* (*FP*) the number of normal behaviours detected as malicious activities.
- *False negative* (*FN*) the number of malicious behaviours detected as normal activities.

False positive rate (FPR) shows the percentage of legitimate instances misclassified as Bot instances.

8 $$ {\text{FPR}} = \frac{\text{FP}}{{\left( {{\text{TN}} + {\text{FP}}} \right)}} $$

Detection rate (DR), also called recall, indicates the percentage of Bot instances that were predicted as Bot instances.

9 $$ {\text{DR}} = \frac{\text{TP}}{{\left( {{\text{TP}} + {\text{FN}}} \right)}} $$

Accuracy indicates the percentage of correct predictions of all instances.

10 $$ {\text{Accuracy}} = \frac{{\left( {{\text{TP}} + {\text{TN}}} \right)}}{{\left( {{\text{TP}} + {\text{TN}} + {\text{FP}} + {\text{FN}}} \right)}} $$

Precision indicates the percentage of instances correctly classified as a positive instance.

11 $$ {\text{Precision}} = \frac{\text{TP}}{{\left( {{\text{TP}} + {\text{FP}}} \right)}} $$

*F*-measure: a measure of a test’s accuracy. It considers both the precision and the recall of the test to compute the score.

12 $$ F{\text{-measure}} = \frac{{\left( {2 \times   {\text{Precision}} \times   {\text{Recall}}} \right)}}{{\left( {   {\text{Precision}} + {\text{Recall}}} \right)}} $$

The results obtained demonstrate that the proposed approach gives the highest detection and accuracy rate with the neural network at around 99 %. The features based on the PCA algorithm gave lower accuracy and detection rates than the other approaches at around 93 and 91 %, respectively, as shown in Table 5.

**Table 5:** Neural network results with CART subset, ReliefF subset and PCA

Classifier	Proposed approach		ReliefF		PCA	
Neural network	Accuracy rate	Detection rate	Accuracy rate	Detection rate	Accuracy rate	Detection rate
	99.20	99.08	97.37	93.77	91.06	93.23

**Fig. 3:**
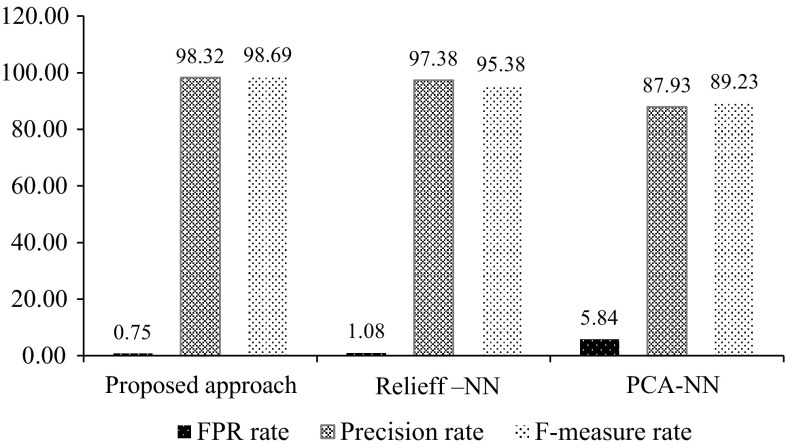
FPR, precision rate and F-measure comparison

Figure 3 gives the FPR, precision rate and F-measure of the Bot detection system using the various feature selection approaches with the same set of TCP features. The results show that the highest average F-measure rate was 98.32 % for the proposed methodology, while the lowest F-measure rate was 87.93 % for the PCA algorithm. Moreover, the proposed approach gave the lowest false positive rate of around 0.75 %.

**Fig. 4:**
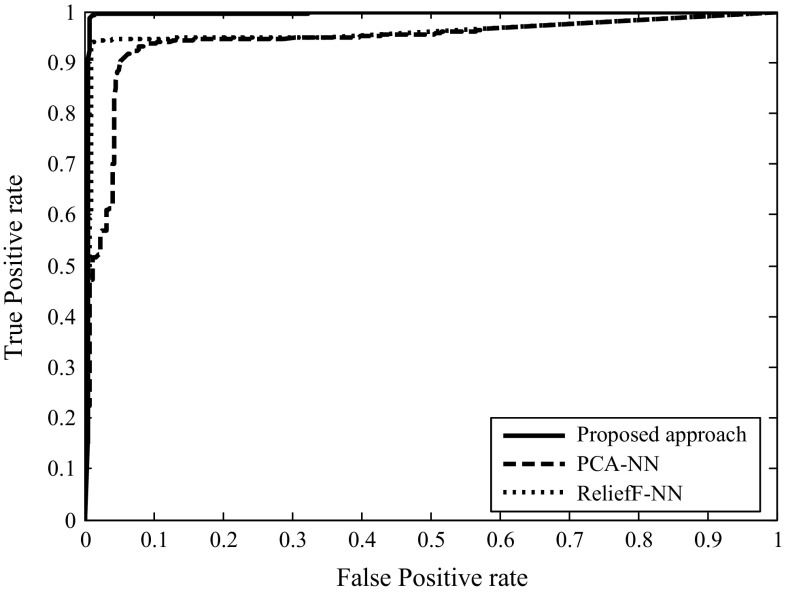
ROC Comparison

To test the efficiency of our proposed approach in detecting P2P Bots, the receiver operating characteristic (ROC) curve is plotted to show the trade-off between TPR and FPR. A perfect classifier would have an area under curve (AUC) close to 1.0. The *x*-axis represents a FPR, and the *y*-axis represents a TPR. As shown in Fig. 4, the area under curve (AUC) is 0.994 for the TCP control packet feature selection based on CART with the neural network. It is found that the proposed approach performs well in classifying P2P Bot connection traffic based on TCP control packets in a 30-s time interval.

The computational time of each feature selection algorithms is estimated to measure the performance of algorithms as expected in actual situation deployment. Also, we measure the neural network computational time that required to train it per second based on the subset of features that obtained from features selection algorithm. These analyses were conducted on 3.00 GHz Intel i7 running Windows 8.1 operating system.

Table 6 provides a comparison of all the features selection algorithm. The decision tree (CART) achieves the minimum time on selection subset of features than other technique using the same dataset as shown in Table 6.

**Table 6:** Features selection algorithm computation time

Features selection algorithm	CART	ReliefF	PCA
Time (s)	2.5	113	4.9

Table 7 shows the comparison of the build time (training time) of neural network models based on features selection subset. The CART algorithm achieved the best results for neural network training time compared with ReliefF subset and PCA. However, the maximum time is based on the subset of features that obtained from ReliefF algorithm.

**Table 7:** Neural network training time with CART subset, ReliefF subset and PCA

Neural network training time	CART features subset	ReliefF features subset	PCA features subset
Time (s)	23	29	25

The main advantage of the suggested approach is reduction network traffic technique that it reduces 60 % of the input packets and retains high detection rates as well as low false positive rate. The bottleneck of the neural network model for Bot detection is the dimensionality and the size of the dataset considered because the amount of the packets that a detection system requires to scan is very large. Therefore, this study proposes a reduction network traffic approach to reduce the size of network traffic and utilize decision tree to reduce the features dimensionality. Additionally, the feature set used in the proposed system represents connection behaviour that helps to detect Bots in the early phase of their life cycle before they begin malicious activities. Moreover, to bypass the encrypted network traffic, connections-based detection mechanism was designed which utilizes the information in the header of TCP control packets. Thus, it cannot be confused by payload encryption techniques. The performance of the proposed approach was tested using real network traffic and is compared with some of the existing P2P Bot detection techniques.

Compared with other approaches, the proposed approach reduces the amount of processing required to increase performance. It is not easy to compare different Botnet detection approaches because each uses different datasets and Botnet samples in the experiments. Therefore, the proposed approach is compared with another detection approach based on accuracy, detection and false positive rates. Table 8 shows the results of the comparison of our results with those in other published work based on the analysis of network traffic to detect P2P Botnets. The table also demonstrates that the accuracy and false positive rates using the proposed approach are better than those gained by previous solutions. Moreover, due to its design, our solution is able to detect single Bot infections and it is not necessary to associate activity among multiple hosts during the detection phase, as in the case with TAMD [60], BotMiner [39] and BotSniffer [38]. On the other hand, several existing schemes [40, 61] support the detection of individual Bot infections, but they use DPI. In contrast, our solution needs only information about network connections; it does not examine payload content. Therefore, it is immune to Botnets that use encryption methods. However, the present approach can detect P2P Bots and classify the host connections as legitimate or malicious. A limitation of the proposed research is that it only considers TCP traffic to detect Botnet traffic. Therefore, if Botnets use UDP packets for communication, this may not be detected by the proposed approach. In the future, thus proposed approach will be improved to have the ability to discover the Botnets that utilize the UDP protocol for communication.

**Table 8:** Comparison with other published approaches

Approaches	FPR %	Accuracy %
Fedynyshyn et al. [25]	7.8	92.9
Wen-Hwa et al. [24]	0	98
Proposed approach	**0.75**	**99.20**

Bold entries relate to the results achieved by the technique proposed in this paper

There are four principal difficulties in the detection of Botnet behaviour: firstly, the network traffic is continuous, which indicates that it is persistent and features will change over time. Furthermore, Botnets dynamically change via Bot updates or altering their operation in various life cycle stages after receiving instructions from a Botmaster. These phenomena are termed concept drift, and this is currently a serious issue for any detection methods [62]. Secondly, there is always the risk of a new Botnet emerging on a network and spread the malicious behaviour stealthily. Moreover, the behaviour of an infected host might seem like legitimate behaviour, and it is difficult to detect malicious activities if the classifier was not trained for this behaviour previously. Thirdly, evaluating the entire network traffic in real-time is a computationally expensive task due to the speed of network traffic. Finally, the availability of up-to-date Botnet traffic dataset remains a key challenge in detection of Botnets. The universality and precision of the classifier depend on the training datasets quality. The available Botnet dataset is formed in academic experiment source due to the security and privacy issue, and it is hard for the researcher to get a Botnet traces from other such as corporate networks.

## Conclusion and future work

A joint classification and regression tree (CART) algorithm and neural network have been presented to detect P2P Bot connections. The implementation of the CART–NN requires two sequential steps. Firstly, CART is applied to choose the suitable features important in detecting Bots. Secondly, the result of CART features subset is employed to produce the input layer of the neural network. The proposed method is based on features extracted from TCP control packet headers during 30-s connections between two hosts; thus, it can be used to detect P2P Bot without relying on packet payload, IP address, port number and encrypted traffic. The performance of the proposed detection technique is compared with features selection approach like PCA, ReliefF algorithms. Experimental results show that the proposed DT–NN method achieved high accuracy rates with lower false positive rates. In the future, we plan to extend our approach to real-time systems by adding an unsupervised learning approach to select the most relevant features that would further increase accuracy and performance.
